# Comparing the mental effects of interacting with farm animals and walking in a botanical garden

**DOI:** 10.1371/journal.pone.0312021

**Published:** 2024-10-29

**Authors:** Andrea Temesi, Enikő Kubinyi, Ákos Pogány, Boróka Mária Babarczi-Bereczky, Ádám Miklósi

**Affiliations:** 1 Department of Ethology, Institute of Biology, Eötvös Loránd University, Budapest, Hungary; 2 MTA-ELTE Lendület "Momentum" Companion Animal Research Group, Budapest, Hungary; 3 ELTE NAP Canine Brain Research Group, Budapest, Hungary; 4 HUN-REN-ELTE Comparative Ethology Research Group, Budapest, Hungary; University of British Columbia, CANADA

## Abstract

Over the past two decades, farm animal-assisted therapies have become popular. However, the effects of farm animals on healthy people’s mental states have not yet been investigated. In Study 1, we aimed to explore whether positive effects of human-animal interaction (HAI) can be detected in healthy farm volunteers even after short-term (2–3 hours) work with goats and goatlings on a goat farm. We found that the participants’ state anxiety decreased (p < 0.001) while their trust levels increased (p < 0.001) after interacting with goats. Nevertheless, it is possible that time spent in nature alone can have a beneficial effect on well-being. Therefore, in Study 2, we compared the results of Study 1 with those of a similar short-term outdoor treatment, walking in a botanical garden as a visitor. Similar but smaller effects were found for garden visitors. Decreases in anxiety scores and increases in trust scores were more pronounced in farm volunteers (anxiety: 25% vs 13%, p < 0.001; trust: 13% vs 3%, p = 0.002) after the treatments. Overall, the results suggest that the novel experience of interacting with goats and goatlings has a more positive effect on the mental state of healthy humans than walking in a botanical garden. This finding offer a strong foundation for developing animal assisted therapy methods for individuals with short or long term mental problems, but they can also enhance the wellbeing of mentally healthy people.

## Introduction

Positive HAI improves certain aspects of humans’ physiological health and psychological well-being [e.g., 1]. The presence of an animal can provide an alternative source of social support [2] and reduce the negative effects of potentially stressful events [e.g., 3]. Owners may have a more intimate connection to their animal companions because, in this context, they experience a kind of unconditional relationship ("love") that is typically absent from adult human relationships [4]. Women may perceive children and pets as playing partly similar roles in the family [5]. Companion animal keeping, especially dog keeping, can have a positive effect on the frequency of human social interactions and owners’ physical activity [e.g., 6–8].

The positive effects of HAI have been measured both physiologically and psychologically. After positive human-animal interaction, the concentrations of plasma and urinary oxytocin increased [e.g., 9,10], blood pressure and heart rate decreased [e.g., 11] in humans, in parallel to decreased state anxiety (measured by the Spielberger’s state anxiety inventory—STAI) [e.g., 12–14].

In practice, HAI is often used to achieve therapeutic goals. For example, The Delta Society, a leading international non-profit organisation, differentiated and defined two types of HAI within animal-assisted interventions (AAIs) [15,16]. Animal-assisted therapy (AAT) is a goal-directed intervention that is a part of a treatment process. Animal-assisted activities (AAAs) are not directed toward specific therapeutic goals but provide opportunities for motivational, recreational, and/or therapeutic benefits to improve the quality of life. These interventions take place in various environments (hospitals, nursing homes, schools, etc.) [17].

The therapeutic use of dogs is widespread as easy to carry out, independent of location possibility. However, the Delta Society identified more species that can be considered for similar deployments: cats, guinea pigs, rabbits, and farm animals. The involvement of other species is less common compared to the dog, possibly because these species are typically not so frequently available.

Various farm animal-assisted interventions are becoming increasingly popular worldwide [18,19]. Working with the animals, feeding, cleaning, or grooming them can provide the experience of close physical contact, nurturing, accomplishing a task, or coping with a situation. The subjects of studies exploring the effects of HAI are usually people with some form of mental illness, as is the case with research on farm-animal-assisted interventions [e.g., 14,20,21]. Exposure to farm animals during the interventions is regular over an extended period of at least 10–12 weeks [20]. Studies so far have revealed that these interventions have beneficial effects on depression, anxiety, and self-efficacy [e.g. 14,20,21] in psychiatric patients. Still, even in their case, the changes occur only weeks or months after the treatment [14]. Importantly, these effects are stronger if these people are exposed to more complex and challenging work tasks. The effect can be explained by assuming that people suffering from mental disorders may feel vulnerable, have little self-esteem, and be powerless over their lives more often than their healthy counterparts [e.g., 22,23].

In Study 1, we aimed to explore whether positive effects of HAI can be detected in healthy people even after a few hours of work with goats, similar to people suffering from mental disorders. The study was carried out on a goat farm that regularly receives volunteers each spring starting in 2016 to take part in the work around the adult goats and the newborn goatlings. In farm animal-assisted interventions, the effects on the individuals involved in the therapy are more complex than in indoor treatments. During outdoor interventions, due to the natural environment, the participants are exposed to many different stimuli (including colours, sounds and odours) in a diverse environment. This fact forms the basis of the Green Care (or Farming for Health) concept [18]. The term covers many interventions using farm animals, plants, gardens, or the landscape and rests on the positive connection between exposure to nature and human health [20]. In the Green Care Farming, physical activity in a naturalistic setting can also be beneficial [see for review: 24]. Because of rapid urbanisation, many people have little or no contact with nature, and indoor activities dominate their daily lives [25]. However, many studies have confirmed the importance of preserving our relationship with nature as a vital part of the human experience [see for review: 26,27]. There is evidence that parks and other natural environments play a crucial role in general human health and well-being [e.g., 28]. Walking and other nature-related activities relieve mental fatigue [29], improve life satisfaction [30] and cognitive performance [31], and reduce the risk of mental illness [32]. Caring for living organisms can be an essential part of the relationship with nature, and the positive effects of caring for animals especially must be emphasised [33]. Therefore, to reveal the specific contribution of dealing with animals, in Study 2, we compared the psychological effects of two different short-term outdoor interventions: taking care of goats and goatlings on a goat farm as a volunteer (Farm group) and walking in a botanical garden as a visitor (Garden group). Previous research has shown the anxiety-reducing effect of interaction with farm animals [e.g., 21]. We were interested in the specific effects of the human-animal interaction, considering that people in the Garden group might also experience potentially anxiety-reducing effects due to outdoor physical activity [e.g., 34].

At the same time, experimental studies demonstrated that social skills are affected by anxiety and mood (disorders). For example, the performance in recognising facial displays of emotions is weaker among persons with depression and anxiety [e.g., 35]. In healthy (non-clinical) individuals, anxiety is associated with less effective strategies in social problem-solving [36]. Social judgments, trusting a stranger or a positive response to cuteness may also be influenced by mood [e.g., 37,38]. Based on these findings, we expected that the interaction with the farm animals would decrease participants’ state anxiety and, as a result, improve their social skills and social attitude.

Thus, we deployed some well-established tools to measure social skills and social attitudes. As mind-reading is an essential skill in human social interactions reflecting social sensitivity, we applied the "Reading the Mind in the Eyes Test" [RMET; 39] to investigate how well the participant can assess the mental and emotional state of another person based on that person’s facial expression.

Cuteness perception is usually measured by showing baby or companion animal (dog or cat) images to subjects [40,41]. There is evidence that this effect is not limited to infant faces; there are inter-specific preferences, suggesting a common mechanism coding cuteness in human and nonhuman faces [e.g., 42]. Szánthó et al. [43] investigated the emotional attitude of dog owners using cuteness scores of puppy and adult dog photos. Similar to these studies, we asked our participants to rate cuteness using a self-developed series of images of human infants (babies) and dogs (adults and puppies).

Studies on the effect of oxytocin on various behaviours often measure trust by a test called "trust game", a standard risk game in experimental economics [44]. However, we needed a simple test that could be applied easily and quickly in the goat farm or the botanical garden. Thus, we have developed a picture-rating task similar to that measuring cuteness. Participants had to evaluate the trustworthiness of the persons in the photos before and after their treatments.

We hypothesised that after interacting with the goats and goatlings, participants’ performance of inferring about mental states improves; they rate adults as more trustworthy and images of babies and puppies as cuter than before the interaction. Similar but smaller effects were expected for people visiting the botanical garden. Thus, we predicted an interaction effect between the test trial (pre- vs post-treatment) and the type of experience (Farm vs Garden).

## Materials and methods

### Ethics

All procedures were in accordance with the ethical standards of the institutional and/or national research committee and with the 1964 Helsinki Declaration and its later amendments or comparable ethical standards. Participation was anonymous, so the study did not violate respondents’ privacy. Ethical permission for this study was obtained from the Hungarian ethical review board for psychological research "EPKEB: Egyesített Pszichológiai Kutatásetikai Bizottság (United Ethical Review Committee For Research In Psychology)" (reference number: 2016/11 and 2017/19). Written informed consent was obtained from all subjects involved in the study.

### Subjects

Participants were recruited personally at the locations in spring (recruitment period for farm volunteers: 26 March 2016–10 April 2016; recruitment period for botanical garden visitors: 25 March 2017–7 May 2017). At the goat farm the volunteers were recruited by the farm. During the indicated period approx. 100 volunteers arrived, and we offered them the opportunity to participate in the research on site, providing verbal information about its details. Volunteering with farm animals was a first time experience for all participants. Garden visitors were similarly recruited at the location, so the subjects decided to visit the goat farm/botanical garden by themselves without any prior interactions with the researchers.

At the same time, participants were required to meet health-related inclusion criteria to ensure that psychological data were not affected by certain physiological factors. These criteria were introduced verbally to the applicants by the experimenter during the recruitment process. The related questions were part of the questionnaire completed by the participants at the beginning of the experiment. Medication use, pregnancy or breastfeeding, alcohol, drug, and caffeine consumption, smoking, and intense exercise may affect participants’ anxiety and response to treatment [e.g. caffeine consumption: 45; intense exercise: 46]. Therefore, participants were asked to declare that they were healthy, not taking any medication, were not currently pregnant or breastfeeding, and that they had not consumed alcohol or drugs or exercised extensively on the day of the experiment before it. We did not consider smoking and coffee consumption to be exclusionary reasons, but recorded them. Caffeine consumption on the day of the experiment was allowed and included in the statistical analyses. The vast majority of the participants were non-smokers.

The number of participants, demographic, and health characteristics are summarized in Table 1. Group differences in these variables were statistically tested by t-tests, and chi-squared tests; for larger than 2x2 tables, if more than 20% of the cells had expected count less than 5, the result of the Likelihood Ratio test [LRT] was taken into account. We provide mean ± SD of the groups, or the absolute number and percentage of volunteers in each category.

**Table 1:** Demographic and other characteristic data of the participants.

	Study 1 Farm group (N = 63)	Study 2 Garden group (N = 61)
**Age in years** *		nr = 59
	36.81 ± 9.59	41.02 ± 11.77
**Gender**		
Female	49 (77.8%)	39 (63.9%)
Male	14 (22.2%)	22 (36.1%)
**Education**		nr = 59
High school or lower	21 (33.3%)	19 (32.2%)
Postsecondary	42 (66.7%)	40 (67.8%)
**Place of residence**		nr = 59
Rural	4 (6.3%)	1 (1.7%)
(Incorporated) town	22 (34.9%)	15 (25.4%)
County town	2 (3.2%)	1 (1.7%)
Capital	35 (55.6%)	42 (71.2%)
**Marital status** *		nr = 58
Single	16 (25.4%)	18 (31.0%)
Romantic relationship	22 (34.9%)	6 (10.3%)
Married	19 (30.2%)	28 (48.3%)
Divorced or widow	6 (9.5%)	6 (10.3%)
**Housemates**		nr = 58
Living alone	7 (11.1%)	9 (15.5%)
Living together with someone	56 (88.9%)	49 (84.5%)
**Children** *		nr = 58
Has	20 (31.7%)	33 (56.9%)
Does not have	43 (68.3%)	25 (43.1%)
**Pet**		nr = 58
Has	40 (63.5%)	38 (65.5%)
Does not have	23 (36.5%)	20 (34.5%)
**Smoking**		nr = 58
Yes	5 (7.9%)	6 (10.3%)
No	58 (92.1%)	52 (89.7%)
**Caffeine consumption on the study’s day** *		nr = 57
Yes	32 (50.8%)	42 (73.7%)
No	31 (49.2%)	15 (26.3%)

*: significant difference between the two groups in the given characteristic.

nr: Number of responses in cases when not all group members answered the given question.

We found significant differences between the two groups in various characteristics (age: t_120_ = 2.170, p = 0.032; marital status: chi^2^ test: χ^2^_4_ = 10.796, p = 0.013; children (has/does not have): chi^2^ test: χ^2^_1_ = 7.760, p = 0.005; caffeine consumption: chi^2^ test: χ^2^_1_ = 6.633, p = 0.010). In case of the Garden group, the average age was higher. There was a difference in marital status, namely the ratio of married people was higher, but the ratio of participants in a romantic relationship was lower (there was little or no difference in the ratio of singles and divorced/widow) compared to the Farm group. In line with the above, the ratio of parents in the Garden group was also higher compared to the childless participants. Furthermore, in the Garden group the ratio of coffee consumers (on the day of the experiment, before the experiment) was higher compared to non-consumers, while in the farm group, this ratio was roughly half and half.

### Study 1. Interactions with farm goats

#### Procedure

We collected pre- as well as post-treatment data with four psychological tools. Before the treatment, the experimenter provided a general description of the study aim (investigating the effects of human-animal interaction) and procedure. Participants filled in a datasheet including demographic and health questions (see Table 1). During volunteering, they spent time/worked together with the people they arrived with, i.e. their family members/friends. The pre- and post-treatment data collection (filling in simple paper-and-pencil tests) took roughly 15–20 minutes both times, and the participants completed the tests individually. After the post-treatment data collection, they could choose a refreshment (soft drink) and sweets from a basket offered by us to express our gratitude.

The treatment: The volunteers took part in the feeding and grooming of the animals and the socialising of goatlings for 2–3 hours. The farm staff took care of the safety of the volunteers, who were all given more manageable tasks. Most of the time, they were petting and feeding the goatlings. Of the adult animals, only the friendliest, gentlest animals were encountered. We did not limit the number of animals they could interact with. To make the situation natural, we did not limit the participants’ contact with others either.

Psychological tools:

1. Spielberger’s State Anxiety Inventory (STAI) is a brief, easy-to-administer self-report measure that is widely known in research and clinical practice [47; Hungarian version: 48]. The total range of scores: 20–80.
2. The Reading the Mind in the Eyes Test [RMET; 39; Hungarian version: "Szemekből Olvasás Teszt", SZOT, 49] consists of a series of photos of the eye-region of the face displayed by different actors and actresses. Participants were asked to choose which of the four listed words best describes what the person in the photo is thinking or feeling. The score was based on the number of correct answers. This test originally consists of 36 photos. To avoid repetition, photos were divided into two sub-sets (RMET 1 and RMET 2) for our pre- and post-treatment measurements. Both sub-sets consist of easy and difficult items as well based on a pilot study (N = 87) to circumvent possible ceiling effects. Based on a semi-random disposition, half of the participants were first tested with RMET 1 (pre-treatment) followed by RMET 2 (post-treatment). In contrast, the other half of the participants were tested in a reversed order (first RMET 2 then RMET 1). The total range of the scores: 0–18.
3. The Cuteness Rating Task (CRT) [based on, e.g. 40,43] involves rating human baby and dog photos. Participants were asked to look at pictures in a photo album and to assess the cuteness of each picture. The total range of the scores: 0–160 for each type of picture set (baby and dog photos).
4. The Trust Rating Task (TRT) included portraits of women and men, and participants had to indicate to what degree they agreed with the statement about the trustfulness of the person in the picture. The total range of the scores: 0–160.

Both in CRT and TRT, two completely different sets of photos were applied to control for the effect of a specific set of photos. The participants were assigned one or the other set based on a semi-random disposition. To compare the pre- and post-treatment ratings, after the treatment, the participants received the same set as before, but the photos were in a different random order in the album. The subjects started with one order or the other based on a semi-random disposition. The participants rated the photos one by one. They assessed their agreement on a continuous scale with the statement that the baby or dog in the picture is cute. Continuous rating scale is an alternative to the frequently used Likert-type scales with a limited number of choices. This type of measurement requires participants to express their opinion visually, i.e. to place a mark at an appropriate position on a continuous line. Although manual measurement of respondents’ answers on a sheet of paper is time-consuming, it provides a finer resolution of responses. In addition, the second assessment of the participants is less affected by their previous assessment. Finally, averages were calculated from the scores given to each of the photos so that each participant received a mean baby cuteness, a mean dog cuteness and a mean trust score, both before and after the treatment.

All photos of the babies and dogs for the CRT were sent by people from the authors’ acquaintanceships. The photographers declared in writing that the child or dog in the photo belonged to them, and they gave consent to use the photos for testing and publication purposes. The photos of the adult persons are owned by the Department of Ethology, Eötvös Loránd University. Each photographed person contributed to the use and publication of the images for scientific purposes.

#### Statistical analysis

To demonstrate the accuracy of manual measurement of scale lengths in CRT and TRT, we checked the inter-rater reliability. A random subsample of the subjects’ ratings (20%) has been evaluated by two independent raters (authors AT and BMBB). The result showed perfect inter-rater reliability as Cronbach’s alpha was 1.000, p < 0.001.

We analysed STAI, RMET, Baby CRT, Dog CRT, and TRT scores as separate dependent variables. Analyses of the residuals confirmed normal distribution for Baby CRT, Dog CRT, and TRT variables. In these cases, the treatment effect was analysed in linear mixed models (LMMs). For the data that did not fit the criteria of normal distribution (STAI and RMET variables), we used generalised linear mixed models (GLMMs) with Poisson distribution. The initial models of STAI, RMET, Baby CRT, Dog CRT, and TRT (separate dependent variables) included trial (pre- and post-treatment) as a fixed effect and participant ID as a random term. Testing the effect of the trial was the focus of our analysis, as a significant effect indicates that participants had different scores before and after treatment. In addition to the above variables, the possible confounding effects of participants’ sex, age, educational level, marital status, whether he/she is living alone or with someone, whether he/she has (a) child(ren), whether he/she has (a) pet(s), caffeine consumption and photo sets have been tested using stepwise regression with backwards elimination; the final models included only significant effects. Model assumptions have been tested before the analysis. All analyses were conducted in SPSS v22.0.

### Study 2. Comparisons between participants of Study 1 and visitors of a botanical garden

#### Procedure

The procedure for the garden visitors was the same as in Study 1; only the treatment differed. Before the treatment, the experimenter provided a general description of the study aim (i.e. investigating the effects of spending time in nature) and procedure. The participants walked with the people they arrived with, i.e. their family members/friends. Similar to Study 1, data collection was conducted in spring.

The treatment: The participants walked around and looked around the botanical garden ("Soroksári Botanical Garden", Budapest, Hungary). With 60 acres of diverse habitats, the garden offered a meaningful pastime for 2–3 hours. To make the situation natural, we did not limit the participants’ contact with others. Of course, just as the farm volunteers could ask for help from the farm staff if it was necessary, the staff of the botanical garden were also available to help and inform visitors when needed.

#### Statistical analysis

We performed the analysis similarly to Study 1, but in the case of Study 2, testing for the trial x group interaction effect was the focus of our analysis, as a significant interaction indicates that the effect of experience was different between participants involved in interacting with goats and those who were not. Analyses of the residuals confirmed normal distribution for Baby CRT, Dog CRT, and TRT variables. In these cases, the treatment effect was analysed in linear mixed models (LMMs). For the data that did not fit the criteria of normal distribution (STAI and RMET variables), we used generalised linear mixed models (GLMMs) with Poisson distribution. The initial models of STAI, RMET, Baby CRT, Dog CRT, and TRT scores (separate dependent variables) included group (farm vs garden) and trial (pre- and post-treatment) and the two-way interaction between them as fixed effects and participant ID as a random term. Relationships between variables were investigated using Pearson correlations. All analyses were conducted in SPSS V22.0.

## Results

### Study 1. Interactions with farm goats

**Fig 1:**
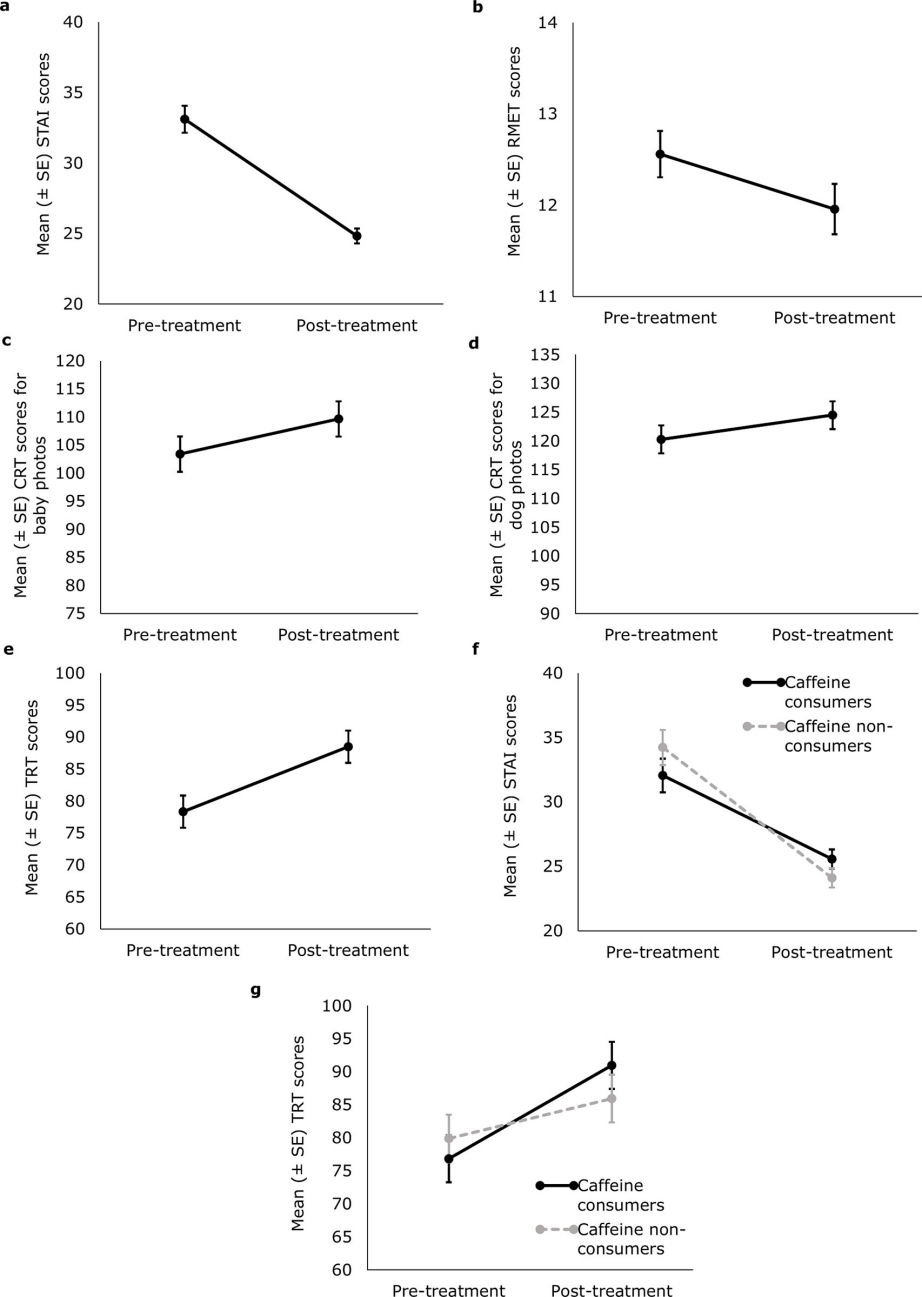
Mean (± SE) pre- and post-treatment scores of the Farm group. a) Spielberger’s State Anxiety Inventory (STAI), b) Reading the Mind in the Eyes Test (RMET), c) Baby Cuteness Rating Task (Baby CRT), d) Dog Cuteness Rating Task (Dog CRT), e) Trust Rating Task (TRT), f) STAI—caffeine consumption, g) TRT—caffeine consumption.

We found a significant effect of the treatment for all variables (Fig 1). Participants had lower anxiety scores (F(1,124) = 88.750, B = -8.279 ± 0.879, t(124) = -9.421, p < 0.001; Fig 1A) and RMET scores (F(1,124) = 4.684, B = -0.603 ± 0.278, t(124) = -2,164, p = 0.032; Fig 1B) after the treatment compared to pre-treatment. During the evaluation of the photos, higher scores were given after treatment than before treatment in both Baby CRT (F(1, 62) = 14.191, B = 6.242 ± 1.657, t (62) = 3.767, p < 0.001); Fig 1C), Dog CRT (F(1, 62) = 11.328, B = 4.225 ± 1.255, t (62) = 3.366, p = 0.001; Fig 1D) and TRT (F(1, 62) = 32.077, B = 10.159 ± 1.794, t (62) = 5.664, p < 0.001; Fig 1E).

Among demographic and other confounding variables, caffeine consumption also had a significant effect on changes in anxiety (effect of caffeine consumption x trial interaction: F(1, 122) = 4.587, B = 3.638 ± 1.699, t (122) = 2.142, p = 0.034; Fig 1F) and trust levels (effect of caffeine consumption x trial interaction: F(1, 61) = 5.472, B = 8.106 ± 3.466, t (61) = 2.339, p = 0.023; Fig 1G). Caffeine consumers had lower anxiety scores before volunteering compared to non-consumers. However, the change in their scores was less pronounced. A different pattern was observed for trust scores: although caffeine consumers initially had lower scores than non-consumers, their scores increased more after treatment.

### Study 2. Comparisons between participants of Study 1 and visitors of a botanical garden

**Fig 2:**
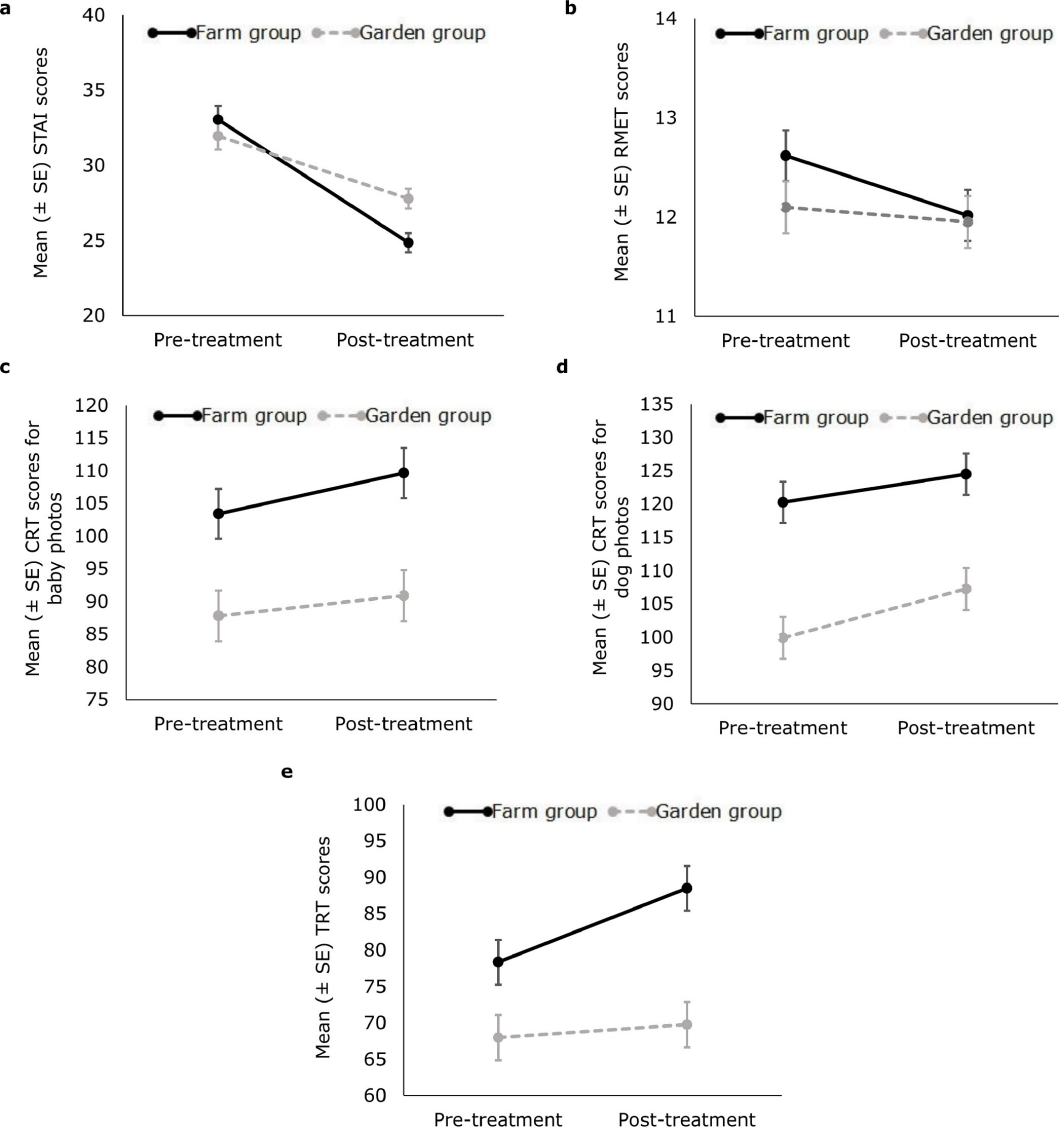
Mean (± SE) pre- and post-treatment scores of the Farm and Garden group. a) Spielberger’s State Anxiety Inventory (STAI), b) Reading the Mind in the Eyes Test (RMET), c) Baby Cuteness Rating Task (Baby CRT), d) Dog Cuteness Rating Task (Dog CRT), e) Trust Rating Task (TRT).

First, we investigated how the different experiences on the farm and the botanical garden affected anxiety. The mean pre-treatment anxiety in the Farm- and Garden group was similar (Farm-group vs Garden-group: M ± SD = 33.24 ± 7.90 vs 32.08 ± 6.96). Regarding different changes between groups, the Trial (pre-and post-treatment) x Group (Farm and Garden) interaction for anxiety was statistically significant. Although anxiety scores in both groups decreased from pre-treatment to post-treatment (effect of Trial: F(1, 244) = 123.363, B = -4.173 ± 0.791, t (244) = -5.277, p < 0.001), this decrease was more pronounced in the Farm-group (effect of Trial x Group interaction; change of anxiety scores from Pre-treatment to Post-treatment trials, Farm group vs Garden group: F(1, 244) = 13.049, B = -4.023 ± 1.114, t (244) = -3.612, p < 0.001; Fig 2A). The demographic variables and other distractors did not affect anxiety and were excluded from the final model.

We found no effect of the treatment (main effect of Trial) or whether this included HAI or not (Trial x Group interaction) in the Reading the Mind in the Eyes Test (effect of Trial x Group interaction: F(1, 244) = 1.286, p = 0.258; effect of Trial: F(1, 246) = 3.128, p = 0.078; effect of Group: F(1, 245) = 0.857, p = 0.355; Fig 2B).

The Trial x Group interaction in the Cuteness Rating Task was not significant and thus was excluded from the final model. However, both Trial and Group had significant main effects. In both groups, participants had higher CRT scores after the treatment, and the participants in the Farm group had higher scores than the participants in the Garden group both before and after the treatment (CRT scores of baby photos: effect of Trial x Group (before exclusion): F(1, 122) = 1.227, p = 0.270; effect of Trial: F(1, 123) = 11.083, B = 4.704 ± 1.413, t (123) = 3.329, p = 0.001; effect of Group: F(1, 122) = 10.567, B = -17.193 ± 5.289, t (122) = -3.251, p = 0.001; CRT scores of dog photos: effect Trial x Group (before exclusion): F(1, 122) = 2.331, p = 0.129; effect of Trial: F(1, 123) = 31.439, B = 5.760 ± 1.027, t (123) = 5.607, p < 0.001; effect of Group: F(1, 122) = 19.201, B = -18.811 ± 4.293, t (122) = -4.382, p < 0.001; Fig 2C and 2D).

In the Trust Rating Task, the effect of the Trial x Group interaction was statistically significant. Although the scores in both groups increased from pre-treatment to post-treatment (effect of Trial: F(1, 122) = 20.616, B = 1.777 ± 1.874, t (122) = 0.948, p < 0.001), this increase was more pronounced in the Farm group (effect of Trial x Group interaction; change of TRT scores from Pre-treatment to Post-treatment trials, Farm group vs Garden group: F(1, 122) = 10.167, B = 8.382 ± 2.629, t (122) = 3.189, p = 0.002; Fig 2E). The participants in Farm group had higher scores than the participants in Garden-group both before and after the treatment (effect of Group: F(1, 122) = 12.121, B = -18.745 ± 4.382, t (122) = -4.278, p = 0.001; Fig 2E). The demographic variables and other distractors had no effect on TRT scores and were excluded from the final model.

Analyses of the interdependence of our various response variables (with treatment groups combined) revealed a weak correlation of anxiety and RMET scores with other variables (all r < 0.17). However, positive correlations were found between CRT scores for baby photos and CRT scores for dog photos (r = 0.35, p < 0.001). Furthermore, CRT scores for dog photos and TRT scores positively correlated (r = 0.34, p < 0.001), and this positive correlation was even more pronounced between CRT scores for baby photos and TRT scores (r = 0.61, p < 0.001).

## Discussion

In Study 1, we investigated the effect of interacting with goats on short-term changes in human well-being under field conditions. In Study 2, our aim was to compare the additional psychological benefits associated with taking care of goats as opposed to walking in a botanical garden. In both situations, participants were outdoors and performed mild physical activity. In order to keep the situation natural, we did not limit the number of people with whom the participants could interact during the treatment, but at the same time, there was no relevant difference between the two groups (Farm group and Garden group) in human interaction possibilities, because in both groups the participants spent time in the company of their friends or families, with whom they arrived. Due to the similar natural and relaxing environment, we expected a beneficial impact in both groups, but interacting with animals was expected to result in a greater decrease in anxiety and a higher elevation of social skills and attitudes.

The Spielberger’s State Anxiety Inventory (STAI) revealed that anxiety scores decreased with treatment in both groups. However, the perceived anxiety of the farm volunteers decreased to a greater degree as a result of goat caretaking compared to the anxiety changed due to walking in the botanical garden. Yoga [50], music [51], and various relaxation and meditation techniques [52], among others, also have the potential to relieve the mental and physical tension caused by everyday stress. In most studies, a measurement of state anxiety is used for confirmation. The relaxing effect of a single treatment was, in general, between 6 to 33% reduction in post-treatment values in healthy populations [53–56]. The effects in our study fit into this range: there was an overall 25% decrease in the Farm group and a 13% decrease in the Garden group as a result of the treatment. The more marked reduction in the anxiety level of the Farm group in comparison to the Garden group supports our hypothesis about the specific effect of farm animals. There are multiple, non-mutually exclusive explanations for this effect regarding the possible underlying mechanisms.

Working with animals requires and captures attention. Thus instead of self-focused attention, participants focus on observing the animals’ behaviour, including communicative signals. Decreased self-focused attention and mental concentration on the task can lead to the emergence of a flow-like state [57,58] that might have a role in the reduction of stress and anxiety levels [59–61].

At the hormonal level, the activation of the oxytocin system may be a common underlying and core mechanism for most of the positive effects of HAI [reviewed by 62]. Regular eye contact and tactile interaction with the animal subjects might have activated this system [e.g., 9], which exerted an additional effect on reducing anxiety in the Farm group.

From a broader, evolutionary perspective, other factors may also contribute to the effects of HAI. The *Biophilia Hypothesis* describes humans’ tendency to affiliate and maintain contact with nature [63]. As shown in Table 1, participants were typically city dwellers in both groups. It is important to note that in Hungary, people living in villages or incorporated towns tend to work in nearby cities, especially in the vicinity of the capital. Thus, those participants who live in villages and smaller towns are also exposed to the effects of the metropolitan way of life and have little or limited contact with nature. The animals and the farm work can provide links toward creating interaction and developing caring relationships with the natural world [64]. The biophilia concept also explains why the feeling of closeness to animals may activate the oxytocin system [4,65].

In the Reading the Mind in the Eyes Test (RMET), there was a moderate decrease in the performance of both groups. This task was found to be more difficult, requiring higher concentration from the participants; thus, the decrease in performance is probably due to the fatigue of the participants [66,67].

Regarding the Cuteness Rating Task (CRT), participants in both groups obtained higher scores after their respective experiences. However, the mean values of the Farm group were higher both before and after the treatment. We made similar observations in the Trust Rating Task (TRT). Thus, Farm group volunteers had higher values in the cuteness and trust measurements even before encountering the animals. These findings may indicate higher baseline sociability, openness, and/or a more positive attitude and higher overall expectations from participating in the study than in the Garden group. Volunteering with farm animals was a first-time experience for our subjects. Participants reported their excitement when they arrived, they were looking forward to meeting the animals, so they had positive expectations, they were in a positive, expectant mood. As it is well-documented, social evaluations can be affected by mood [37,38]; the overall more positive mood of the participants may, therefore, might provide an explanation for the more positive attitude of this group. The visitors to the garden did not and could not expect such a special experience as petting goatlings–which is definitely considered a curiosity for a city person–but based on their feedback, they also had a pleasant time in the garden.

Importantly, in parallel to higher anxiety reduction, people’s trust scores in the Farm group increased more due to the treatment than in the Garden group, so in this task, we found a differential effect of the treatments. Across-species effects have been found in the case of baby schema: baby-like physical features elicit caregiving from adults. This effect is not limited to human infant faces, as dog or cat faces are found similarly cute [e.g., 34]. For farm volunteers, a similar effect to the above mentioned across-species effect can be seen: members of the group interacted positively with animals, but this positive interaction influenced their assessment of the reliability of human individuals. We assume that interaction with the goats resulted in the activation of gregarious behaviour in the participants compared to the people walking in the botanical garden. This phenomenon can be labelled as social sensitisation, and it seems to manifest in increased social preferences and social intimacy (trust test). It seems that the effect of interacting with animals is superior in trust enhancement to the effect of walking in a botanical garden. Thus, it can be assumed that farm work can also be an effective therapeutic method for people with social phobia or other personality and mental disorders associated with low trust [68–71].

Among the potential confounding variables, caffeine consumption had an effect on the change of farm volunteers’ anxiety and trust scores. Caffeine consumers had lower anxiety scores prior to volunteering than caffeine non-consumers. However, the scores of the former group also changed to a lesser extent. A different trend was observed for trust scores. Caffeine consumers had lower pre-treatment scores than caffeine non-consumers, but their post-treatment scores were higher. Of the 63 participants, 32 consumed caffeinated beverages on the day of the experiment before it. All but three people drank coffee. The vast majority of them did it shortly before the experiment, in the 4–5 hours prior to it. There are consistent findings [see for review e.g. 72] for the positive effect of caffeine on mood, which may explain the lower pre-treatment anxiety scores and higher post-treatment trust scores of caffeine consumers. The results certainly indicate that caffeine consumption by research participants should be taken into account when measuring anxiety and various social assessments. When comparing the Farm and Garden groups, the effect of caffeine consumption was no longer detectable, which is probably because while there were almost equal numbers of caffeine consumers and non-consumers in the farm group, this proportion shifted in the garden group: the number of consumers was nearly three times the number of non-caffeine users.

Moderate or strong positive correlations between the CRT and TRT scores indicate that participants who found the babies and dogs to be cuter tended to trust the persons in the TRT photos better. This was especially true for pictures representing humans, that is, for the CRT baby photos and TRT photos. The correlation may be explained by overlapping neural structures underlying cuteness perception and trustworthiness assessment [73–76]. At the same time, it may also be due to individual differences in use of scales, that is, those subjects who gave high cuteness ratings also used the higher end of the trust scale.

### Limitations

Important limitations for the interpretation of the results are that the research occurred in the field and under less controlled conditions. We did not limit the social interactions of the participants, which could have affected their mood and thus also their evaluations in the test tasks. Participants arrived in small groups (family or friends). Although the responses and measurements were taken individually, within-group similarities (e.g., due to shared genetic/social background in the family, or personality/temperament similarities between friends) remained unexplored by design or statistically in our study. To be able to address this source of variation, an increased number of volunteers, hence sample size, are needed in the future. The treatments were short-term (2–3 hours), so we did not examine the long-term or regular effects of interacting with goats or walking in the garden. This was a between-subject study, so we cannot investigate the possible effect of pre-existing attitude differences between the members of the two groups. At the same time, we consider it important that even in the less controlled, spontaneous natural conditions described above, there was a detectable difference between the two groups in the variables (anxiety, trust) measured before and after the treatment.

In future research, the next step would be to check the reproducibility of the results using a full within-subject design with participants being allowed once to interact with goats and once not (walking or carrying out different activities on the farm) in random order.

Farm exposure may be especially effective in people with more positive attitude and positive, expectant mood. Future research using a cross-treatment design with subjects having adequate or incompatible preferences or attitudes could provide more support to this assumption. For example, specifically pre-selected people based on their preference for animals and plants should be exposed to both situations. We suggest that the effectiveness of animal-assisted therapies could be improved by including the most responsive participants or patients, that is, those who have the most positive attitude towards the given animal species. Future studies should also aim to establish the potential long-term effects of such exposure in healthy subjects, as both healthy children/adults and children/adults living with some mental problems may benefit from such an experience.

## Conclusions

We assumed that anxiety levels, social skills, and social attitudes change differently in farm visitors and garden visitors due to the presence or lack of interaction with animals. We found that anxiety and trust changed differently in the two groups. Farm visitors experienced a greater reduction in anxiety levels and a greater increase in trust levels than garden visitors. However, farm visitors expressed a more positive attitude towards social beings even before the visit, as revealed by the pre-treatment cuteness and trust test. It is possible that this more positive attitude contributed to the difference between the two groups. Despite this limitation, we found evidence that the Green Farm Concept can contribute to anxiety reduction and trust increase in healthy participants and not only in people suffering from mental health issues. Exploring the therapeutic possibilities for people living with some form of mental illness and investigating the relaxation and prevention possibilities for healthy individuals are similarly essential [e.g. 77,78].

## Supporting information

S1 Dataset The data sets supporting this article have been uploaded as part of the supplementary material. (XLS)
